# *Helicobacter pylori* associated primary cutaneous nodular amyloidosis improvement through debulking and cauterization

**DOI:** 10.1016/j.jdcr.2024.04.033

**Published:** 2024-04-30

**Authors:** Victoria Garfinkel, Heather Woodworth Goff

**Affiliations:** a Medical School, The University of Texas Southwestern Medical Center, Dallas, Texas; b Department of Dermatology, The University of Texas Southwestern Medical Center, Dallas, Texas

**Keywords:** *Helicobacter pylori*, *Helicobacter pylori* with cutaneous involvement, primary cutaneous amyloidosis, primary cutaneous nodular amyloidosis, primary cutaneous nodular amyloidosis and infection, primary cutaneous nodular amyloidosis and treatment

## Introduction

Amyloidosis encompasses a range of conditions characterized by the abnormal extracellular accumulation of amyloid protein, forming nonfunctional beta-pleated sheets and manifesting as either localized, cutaneous, or systemic disease.^1^ When amyloidosis involves the skin without evidence of systemic involvement, it is termed primary localized cutaneous amyloidosis. This disease is generally further divided into 3 subtypes: macular, lichenoid, and nodular. Nodular amyloidosis, derived from immunoglobulin light chains (amyloidosis light chain proteins) produced by plasma cells infiltrating the skin without associated plasma cell dyscrasia, is the least frequent subtype with only 60 cases reported.^1^^,^^2^ Literature suggests possible associations between primary cutaneous nodular amyloidosis (PCNA), autoimmune disease, and trauma.^2^^,^^3^ Stigall et al reported an association between infection and PCNA.^3^ Herein, we report the second case of PCNA associated with an infection, specifically *Helicobacter pylori*.

## Case report

A 58-year-old Asian male with no significant past medical history presented with multiple, waxy erythematous to brown, plaques on bilateral cheeks ranging in size from 1.0 × 0.9 cm to 4 × 3 cm that were present for 3 years. Biopsy revealed diffuse deposition of pink amorphous material from the upper papillary dermis throughout the dermis that stained positive with congo red. Immunohistochemistry staining displayed kappa and lambda expression in plasma cells. Mass spectrometry analysis of the Congo red-stained lesions detected a peptide profile consistent with AL (lambda)-type amyloid deposition, confirming a diagnosis of PCNA. Multiple treatments were attempted, including intramuscular and intralesional methotrexate and triamcinolone, oral minocycline, topical tacrolimus, oral colchicine, and infrared light, but only resulted in slight flattening of the lesions without significant improvement. Subsequently, we decided to treat with serial shave debulking and hyfrecation in stages over a 14-month period which proved satisfactory.

Systemic evaluation revealed normal serum protein electrophoresis, urine protein electrophoresis, and kappa/lambda ratio. However, serum free kappa light chains were elevated. Quantitative immunoglobulin assay showed normal levels of IgG, IgM, and IgE, but elevated IgA. Additionally, we tested for other potential factors influencing plasma cell antibody production, including presence of antinuclear antibody, C3 or C4 elevation, and infections like tuberculosis, *H pylori*, Lyme disease, or hepatitis B/C. Results were negative or normal for all except *H pylori*. A positive breath test prompted a gastrointestinal referral, leading to the discovery of stomach ulcers. Treatment for *H pylori* eradication was successful.

## Discussion

The precise mechanism of amyloid deposition in PCNA remains uncertain, and diagnosis can be difficult, as 40% of primary systemic amyloidosis cases will have cutaneous findings that are clinically and histologically identical to PCNA.^3^ In this case, we suspect that the *H pylori* infection was associated with the development or at least contributed to the atypical, bilateral, and symmetric presentation of PCNA. Proteins associated with *H pylori* activate phagocytes and lymphocytes, resulting in chronic antigenic stimulation.^4^ Gastric mucosa-associated lymphoid tissue lymphoma, a condition associated with *H pylori*, has also been linked to monoclonal gammopathy of undetermined significance, Waldenstrom disease, and multiple myeloma. These associations are not surprising, as mucosa-associated lymphoid tissue also contains plasmacytes that can be stimulated by *H pylori* antigens.^5^ A case report of primary systemic amyloidosis secondary to multiple myeloma with an associated *H pylori* infection, displays a potential linkage between *H pylori* and amyloid deposition.^6^ Although primary systemic amyloidosis and PCNA are distinct entities, their histologic and clinical overlap suggests potential similarities in causation, despite PCNA remaining localized to cutaneous tissue. Additionally, infections with the spirochete organisms, such as *Borrelia* and *Treponema pallidum* are associated with primary cutaneous B-cell lymphoma. Cutaneous lesions of syphilis are also plasma-cell rich.^7^ The relationship between these infections, plasma cell overproduction, and chronic antigenic stimulation can be used to rationalize the deposition of immunoglobulin light chain that was found in the skin of this patient, leading to the PCNA diagnosis. The detection of elevated kappa light chains and IgA in our patient suggests the presence of chronic antigenic stimulation, further supporting an association between the atypical PCNA presentation with *H pylori* infection. Consequently, it is reasonable to screen for infectious sources of antigenic stimulation and antibody production in patients diagnosed with PCNA with a normal serum protein electrophoresis and elevated immune markers.

Following unsuccessful responses to other medications, we decided to use serial shave with a dermablade and hyfrecation to debulk the lesion, a treatment approach similar to that for rhinophyma. To minimize risk of scarring, we applied principles from facial resurfacing techniques, noting that scarring occurs if resurfacing penetrates deep into the reticular dermis, particularly at the insertion point of the arrector pili muscle to the bulge region of the hair follicle.^8^^,^^9^ Preservation of the bulge region is essential, as it contains stem cells that serve primary roles in wound healing and reepithelization.^8^^,^^9^ The patient underwent multiple sessions of debulking with cauterization, achieving cosmetic satisfaction. Each session the lesions were anaesthetized with local injection of lidocaine, then shaved with a dermablade and hyfrecated on a low setting of 2, until the papular component was flat and even with surrounding tissue. The left cheek required 4 sessions, while the right cheek required 3. Sessions were spaced at 3-month intervals to assess improvement and allow for healing. Once a satisfactory cosmetic result was achieved, a single session of pulse-dye laser therapy was used to reduce visible ectatic vessels with a 7-mm spot, 40-ms pulse width and 12 J of power for larger vessels. This was followed by a second pass of 6 ms and 8 J for background erythema. Six months after the completion of all treatments the patient remained clear of new lesions and free from relapse of *H pylori*.

**Fig 1:**
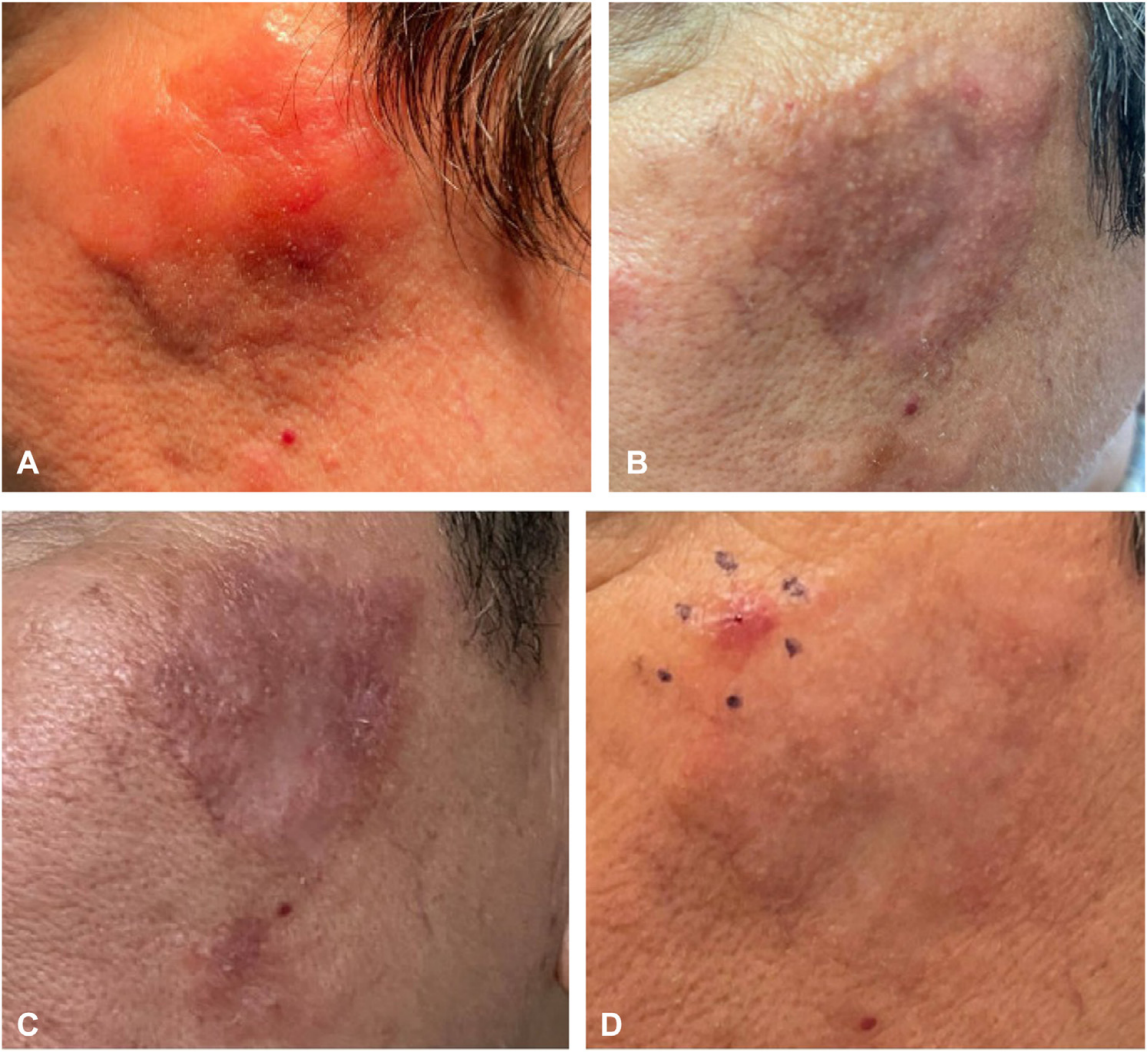
Primary cutaneous nodular amyloidosis on *left* cheek depicting progression of serial shave debulking and hyfrecation treatments. Pretreatment presentation with an erythematous to *brown* plaque (**A**). After 3 sessions of treatment (**B**). Four sessions at the end of 14-month period (**C**). Seventeen months posttreatment with pen marking recurrent plaque in *top left corner* of image (**D**).

**Fig 2:**
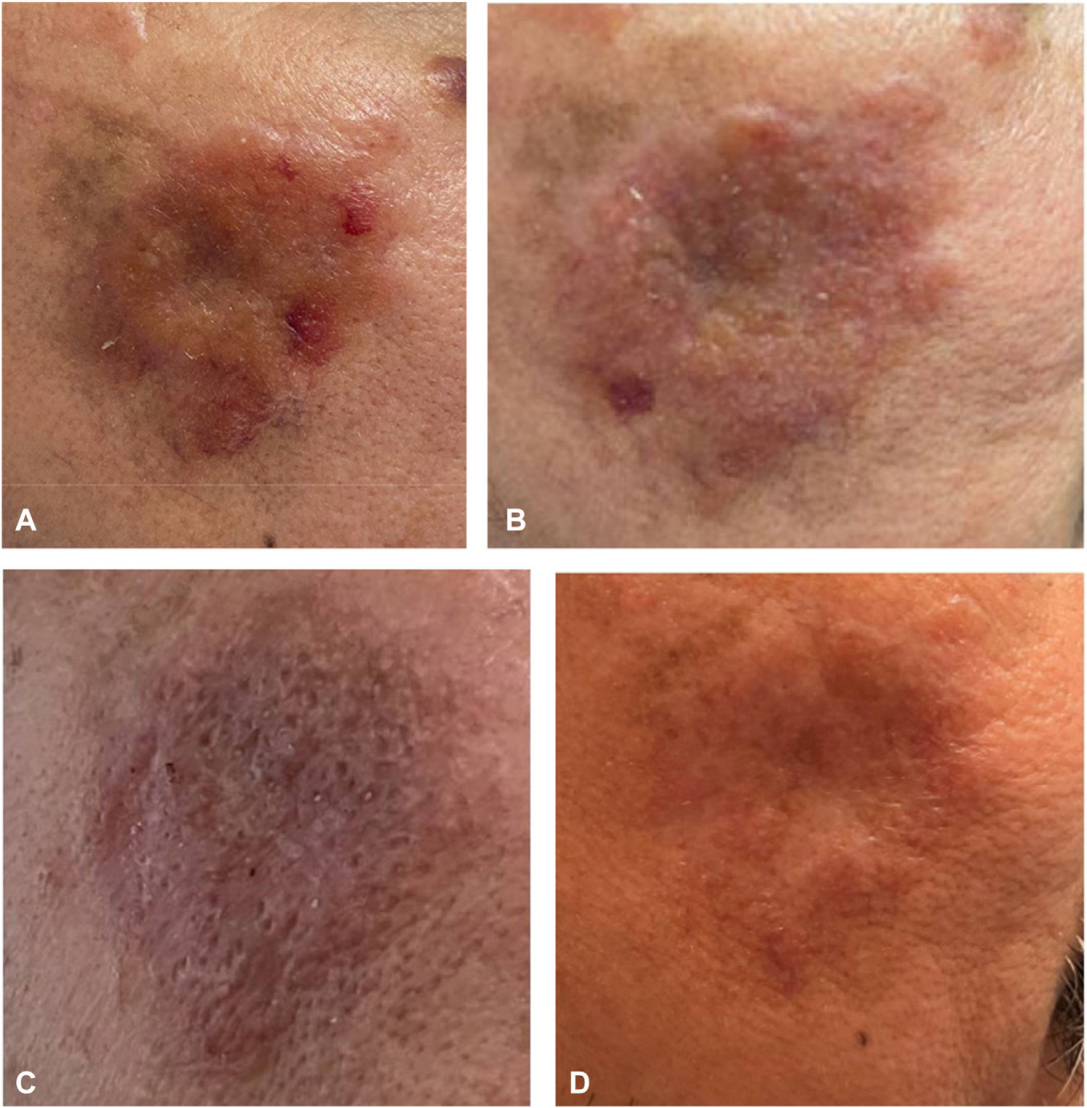
Primary cutaneous nodular amyloidosis on *right* cheek depicting progression of serial shave debulking and hyfrecation treatments. Pretreatment stage presentation with an erythematous to *brown* plaque (**A**). After 2 sessions of treatment (**B**). Three sessions at the end of 14-month period (**C**). No recurrence observed at 17 months posttreatment (**D**).

At 17 months posttreatment, a few small nodules recurred on the left cheek, as seen in Fig 1, with biopsy similar to pretreatment lesions. This recurrence is predictable as amyloid can persist beyond the bulge region, into the reticular dermis and subcutaneous tissue.^10^ However, absence of recurrence on the right cheek may be attributed to complete removal of the lesion or a reduction in chronic antigenic stimulation as seen in Fig 2. This case emphasizes the importance of testing for various antibody-driven processes that may contribute to PCNA, a disease that lacks clear work-up and treatment guidelines in the literature.

## Conflicts of interest

None disclosed.
